# Regulation of human chorionic gonadotropin beta subunit expression in ovarian cancer

**DOI:** 10.1186/s12885-019-5960-2

**Published:** 2019-07-30

**Authors:** Aleksandra Śliwa, Marta Kubiczak, Anna Szczerba, Grzegorz Walkowiak, Ewa Nowak-Markwitz, Beata Burczyńska, Stephen Butler, Ray Iles, Piotr Białas, Anna Jankowska

**Affiliations:** 1 Department of Cell Biology, Poznan University of Medical Sciences, 5D Rokietnicka Street, 60-806 Poznan, Poland; 2 Gynaecologic Oncology Department, Poznan University of Medical Sciences, 33 Polna Street, 60-101 Poznan, Poland; 3 Centre for Investigative and Diagnostic Oncology, Middlesex University, The Burroughs, London, NW4 4BT UK; 4 MAP Diagnostics Ltd, The iLAB, Stannard Way, Bedford, Bedfordshire MK44 3RZ UK; 5 College of Health, Abu Dhabi University, Abu Dhabi, United Arab Emirates

**Keywords:** Human chorionic gonadotropin beta subunit, DNA methylation, Gene expression regulation, AP2 transcription factor, Ovarian cancer

## Abstract

**Purpose:**

Expression of human chorionic gonadotropin beta subunit by cancers is extensively documented, yet regulation of the multiple genes that can code for this protein is poorly understood. The aim of the study was to examine the mechanisms regulating *CGB* gene expression in ovarian cancer.

**Methods:**

Expression of *CGB* genes and *SP1*, *SP3, TFAP2A* transcription factor genes was evaluated by RT-qPCR. The methylation status of *CGB* genes promoter regions was examined by methylation-specific PCR.

**Results:**

mRNA arising from multiple *CGB* genes was detected in both ovarian control and malignant tissues. However, expression of *CGB3–9* genes was shown to be significantly higher in malignant than healthy ovarian tissues. *CGB1* and *CGB2* transcripts were shown to be present in 20% of ovarian cancers, but were not detected in any of the control samples. Malignant tissues were characterized by DNA demethylation of *CGB* promoter regions. In ovarian cancer *CGB* expression positively correlated with *TFAP2A* transcripts level and expression of *TFAP2A* transcription factor was significantly higher in cancer than in control tissues. In contrast *SP3* expression level was significantly lower in ovarian tumours than in control ovarian tissue.

**Conclusions:**

In ovarian cancers increased expression of human chorionic gonadotropin beta subunit is associated with demethylation of *CGB* promoter regions. *CGB3–9* expression level strongly correlates with expression of the *TFAP2A* transcription factor. Presence of mRNA arising from *CGB1* and *CGB2* genes appears to be a unique feature of a subset of ovarian cancers.

## Background

Human chorionic gonadotropin (hCG) is a hormone known for its significant role in sustaining pregnancy and survival of the foetus. In particular, it supports decidualization of the endometrium and embryo implantation. More recent studies suggest that hCG plays an important role in human placental angiogenesis and maternal immunosuppression as well as immunotolerance of the embryo [1–3].

It has also been shown that expression of hCG, in particular its free beta subunit (hCGβ), is detected in 30–60% of tumours of different origin [4]. Many studies have demonstrated that both hCG and hCGβ promote survival of cancer cells via regulation of cell proliferation, angiogenesis, and apoptosis [1–3, 5–7]. Furthermore, hCGβ secretion by a tumour is strongly associated with metastasis, resistance to therapy, which are consequently linked to poor prognosis [7]. Recently it was reported that hCGβ induces transformation of ovarian surface epithelial cells. Specifically the free hCG beta subunit has been shown to regulate the epithelial-mesenchymal transition process and support migration and invasion of cancer cells [8]. *CGB* overexpression by tumours was shown also to promote valculogenic mimicry – a rate-limiting step in metastasis of ovarian cancer [9].

Although the associations between ectopic expression of hCG/hCGβ and carcinogenesis are now established, the exact molecular mechanisms behind regulation of *CGB* genes that encode for this hormone’s beta-subunit remain unclear [10–17].

The beta subunit of hCG is encoded by a group of highly homologous genes (marked *CGB1*–*CGB9*) but the expression of individual *CGB* genes is uneven. Analysis of mRNA derived from placentas and cancer tissues pointed to a twenty fold higher transcriptional activity of *CGB5* compared to *CGB3* or *CGB8* [18, 19].

During pregnancy *CGB* expression is dependent on epigenetic changes as well as expression of tissue-specific transcription factors [10–17, 20]. Studies have shown that, in placental tissues, regions of DNA encoding the *CGB* gene cluster are hypo-methylated [13]. This was also true of choriocarcinoma samples. Moreover it was demonstrated that methylation sensitive allelic polymorphism in *CGB5* gene promoter regions, may be associated with a higher susceptibility for miscarriage [10–12]. Our recent study also confirmed that changes in *CGB* genes expression, caused by deregulation of epigenetic mechanisms, is associated with increased risk of early pregnancy loss [13].

Another aspect of *CGB* expression regulation by the placenta is the dependency on the access to selected transcription factors: ETS-2, SP1, SP3, AP2, OCT3/4, PPARγ, P53 and MTA3 and their binding capacity [20–26]. However, AP2, SP1 and SP3, are believed to be the key regulators of basal *CGB* transcription in placental trophoblast cells [20–26]. In fact, we have previously shown that, during pregnancy, expression of *CGB* genes strongly correlates with expression of the *TFAP2A* gene, encoding the AP2 transcription factor [13].

In this study we examined the hypothesis that, similar to placenta, *CGB* expression by ovarian cancers is associated with methylation alterations of the *CGB* gene cluster, along with changes in the level of the three key transcription factors: AP2, SP1 and SP3.

## Methods

### Sample collection

Ovarian cancer tissue samples were collected from 19 patients treated by surgery at the Department of Gynaecologic Oncology, Poznan University of Medical Sciences, Poland. The histological subtypes of ovarian carcinomas were: 12 serous, 2 endometrioid, 2 undifferentiated, 2 solid appendages, 1 mucinous. UICC histological grading of the subtypes of ovarian carcinomas was as follows: G1, *n* = 1; G2, *n* = 6 and G3, *n* = 12 stage (Table 1).

**Table 1:** Histological subtypes and grading of studied ovarian carcinomas

Grading	Serous	Mucinous	Solid appendages	Undifferentiated	Endometrioid
G1	0	0	0	0	1
G2	5	1	0	0	0
G3	7	0	2	2	1
Total	12	1	2	2	2

A control group of samples consisted of 12 ovarian tissues that lacked cancerous changes, as evaluated by a clinical pathologist’s macroscopic and microscopic examination. These control tissues were obtained from postmenopausal patients who underwent total hysterectomy with additional oophorectomy due to benign myometrial and endometrial lesions.

The study was approved by the ethics review board of Poznan University of Medical Sciences (Resolution No: 748/08) and all patients participated after written informed consent.

Tissue samples obtained during surgery were stored in RNA*later* (Thermo Fisher Scientific, Waltham, MA, USA) at − 80 °C.

### RT-qPCR assays

Total RNA was isolated from 100 to 300 mg of tissue homogenized in 1 mL of TriPure Isolation Reagent (Roche Diagnostics, Mannheim, Germany) according to the manufacturer’s protocol. RNA concentration and quality was determined spectrophotometrically and by eletrophoresis. The material was stored at − 80 °C for further analysis.

One microgram of total RNA was used as template for reverse transcription using the oligo (dT)10 primer and Transcriptor Reverse Transcriptase (Roche Diagnostics, Mannheim, Germany) according to the manufacturer’s protocol.

RT-qPCR assays were designed to assess the expression level of analyzed genes encoding human chorionic gonadotropin beta subunit as well as *SP1, SP3* and *TFAP2A* transcription factors. *CGB* genes were studied in two groups: 1) genes previously considered as pseudogenes: *CGB1* and *CGB2* (*CGB1–2*), and 2) the most transcriptionally active genes: *CGB3/CGB9, CGB5, CGB6/CGB7, CGB8* (*CGB3–9*). Hydrolysis TaqMan probes and primers used in RT-qPCR assays are presented in Table 2.

**Table 2:** Primers (5′ → 3′) and hydrolysis probes for RT-qPCR with the use of SYBR Green and TaqMan assays

	Gene	Forward primer	Reverse primer	Hydrolysis probe
SYBR Green	*SP1*	tgaaggaaggggctcggggg	ccaggggcaaagtgcccaca	n/a
	*SP3*	caggatgtggtaaagtctatgg	cttctcacctgtatgtgttcttc	n/a
	*TFAP2A*	gctgcctcaccagctgtcgg	acagggacacggggcctttct	n/a
	*HPRT*	tgaagagctattgtaatgaccagt	caaatccaacaaagtctggc	n/a
TaqMan	*CGB1–2* ^a^	ggtccgctgactcyggc ^b^	cagcagcagcccctttgac	6FAM-tcactccctgtctcactcccccacg-BBQ
	*CGB3–9* ^a^	gtgtcsagctcacyccagcatccta ^b^	agcagcccctggaacatct	6FAM-ccgaggtytaaagccaggtacacsaggc-BBQ
	*HPRT*	Human HPRT Gene Assay Cat. No. 05 046 157 001 (Roche Diagnostics, Mannheim, Germany)		

^a^ *CGB3–9* and *CGB1–2* hydrolysis probes were designed and manufactured by Tib Molbiol (Berlin, Germany); ^b^ degenerated base code: S-G/C; Y-C/T

RT-qPCR was performed using LightCycler TaqMan Master or LightCycler FastStart DNA Master SYBR Green I kit (Roche Diagnostics, Mannheim, Germany) on a LightCycler 2.0 Instrument (Roche Diagnostics, Mannheim, Germany). Assays were performed according to the manufacturer’s protocols.

PCR efficiencies were calculated from standard curves, which were generated using serial dilutions of a cDNA library obtained from term placenta. Relative expression of genes was normalized against hypoxanthine-guanine phosphoribosyltransferase 1 (*HPRT*) expression. Data was assembled using the software 4.05 dedicated for the LightCycler 2.0.

### Methylation-specific polymerase chain reaction for *CGB* promoter

DNA was extracted from 50 to 300 mg of tissue samples using AxyPrep Multisource Genomic DNA Miniprep Kit (Axygen, Unin City, CA, USA). DNA concentration and quality was determined using NanoDrop ND-1000 spectrophotometer (Thermo Fisher Scientific, Waltham, MA, USA) and by electrophoresis.

One microgram of genomic DNA was converted with bisulfite and cleaned using EpiTect Bisulfite Kit (Qiagen, Hilden, Germany). The prepared material was stored at − 20 °C for further analysis. The quality of the conversion was verified by PCR using methylation independent primers for death associated kinase 1 gene, *DAPK1* (Zymo Research, Irvine, CA, USA) (Table 3).

**Table 3:** Primers (5′ → 3′) and cycling conditions used in MSP

Primer Name	Forward primer	Reverse primer	Annealing	Elongation
CGB1–2_ M	gaaattaagttcgaagtcgc	cctatcaaccataacgatcg	30 s, 51 °C	30 s, 72 °C
CGB1–2_ NM	gtagaaattaagtttgaagttgt	cctatcaaccataacaatca	30 s, 47 °C	30 s, 72 °C
CGB3–9_ M	tgtttagtttgatggtatcgc	atacccgaaacgatcccc	30 s, 58 °C	30 s, 72 °C
CGB3–9_NM	aattgtttagtttgatggtattgt	aaaatacccaaaacaatcccc	30 s, 55 °C	30 s, 72 °C
DAPK	attgggaaggttaaggyggagggaaatttggt	ccccaaacraaacaatccccaaaaccacattccta	30 s, 59 °C	60 s, 72 °C

*M* methylated variant, *NM* nonmethylated variant

The method used for the assessment of methylation level of *CGB* genes was methylation specific PCR (MSP).

The analyzed allelic *CGB* genes were divided into two groups. The first included *CGB3/CGB9, CGB5* and *CGB8* and the second consisted of *CGB1* and *CGB2*.

Primers used in the study were designed to cover the consensus promoter regions of genes *CGB3–9* and *CGB1–2* respectively. Each set of primers consisted of two pairs: one designed to bind the methylated form of the given sequence (CGB1–2_ M, CGB3–9_ M) and the other to bind the non-methylated sequence (CGB1–2_ NM, CGB3–9_NM). Primer sequences and reactions conditions are presented in Table 3.

After amplification, the PCR products were separated by electrophoresis in 2% agarose gel stained with ethidium bromide (0.5 μg/ml). Images were collected and analyzed with G:BOX iChemi, model XL1.4 (Syngene, Bangalore, India).

### Statistical analysis

All data were analyzed using Statistica 13.3 software package (StatSoft, Kraków, Poland). As the data was not normally distributed non-parametric statistics were applied. Associations between variables in the studied groups of ovarian tumours and control ovarian tissues was assessed using Spearman’s rank correlations. The differences between the two groups were analyzed using Mann-Whitney U tests. *p* < 0.05 was considered to indicate statistical significance.

## Results

### Expression levels of CGB genes in ovarian Cancer tissue

The relative amount of *CGB* transcripts was evaluated between control and cancerous ovarian tissue. *CGB* gene cluster expression was analyzed using RT-qPCR in two assays: CGB1–2 and CGB3–9. In the first we examined *CGB1* and *CGB2* genes expression, and in the second, we quantified transcript levels arising from the most transcriptionally active *CGB* genes found in placental and choriocarcinoma tissue – *CGB3*/*CGB9*, *CGB5*, *CGB6*/*CGB7*, *CGB8* [18, 19].

The analysis of relative expression of *CGB* genes, which can encode chorionic gonadotropin beta subunit protein, varied between control and ovarian tumour samples.

**Fig. 1:**
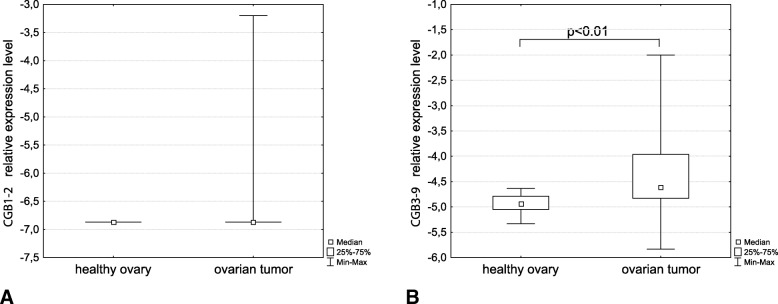
Relative expression level of (**a**) *CGB1–2*, (**b**) *CGB3–9* transcripts in healthy ovary and ovarian tumor tissue measured using RT-qPCR

In the case of *CGB1* and *CGB2* gene transcripts, RT-qPCR assay showed that more than 20% of analyzed ovarian tumours expressed *CGB1–2*, but none of the control ovarian tissues contained detectable levels of these genes transcripts (Fig. 1a).

The CGB3–9 assay detected transcripts in all the control and all ovarian tumours studied. However higher expression was noted for ovarian tumour group and the difference was statistically significant (*p* < 0.01) (Fig. 1b).

The expression level of the analyzed genes did not correlate with either UICC grading or stage of the tumours. The median of *CGB* gene expression level, according to the grade and stage of studied ovarian carcinomas, is presented in Table 4.

**Table 4:** Median of relative *CGB* genes expression level according to the grade and stage of studied ovarian carcinomas

		*CGB1–2*	*CGB3–9*
Grade	G1	0,00E+ 00	1,92E-05
	G2	0,00E+ 00	3,88E-05
	G3	0,00E+ 00	4,70E-05
Stage	I	0,00E+ 00	1,92E-05
	II	0,00E+ 00	6,35E-05
	III	0,00E+ 00	4,40E-05
	IV	0,00E+ 00	6,57E-06

### Methylation of CGB genes promoters in control and ovarian Cancer tissues

Methylation specific PCR was used to establish the methylation patterns of *CGB* gene promoter regions. The allelic *CGB* genes were again divided into two groups. The first assay included *CGB1* and *CGB2* (CGB1–2) and the second one included *CGB3*, *CGB5* and *CGB8* (CGB3–9). Utilization of MSP allowed discrimination of methylated and nonmethylated DNA templates as well as quantification of methylation levels in the studied groups.

**Fig. 2:**
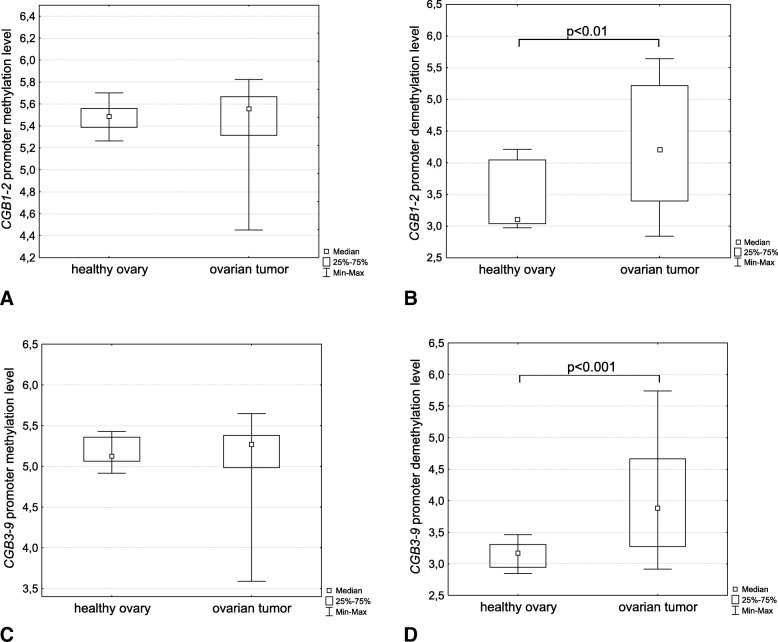
Methylation status of (**a**, **b**) *CGB1–2* and (**c**, **d**) *CGB3–9* promoter sequence in healthy ovary and ovarian tumor tissue analyzed using MSP

Densitometric analysis of electrophoretically separated MSP products obtained from *CGB1–2* and *CGB3–9* promoters demonstrated significant differences between control and ovarian cancer tissues but only in assays targeting demethylated variants of the promoter. Indeed, the use of primers recognizing non-methylated DNA confirmed that ovarian tumour tissues are characterized by a profoundly higher demethylation level in case of both *CGB1–2* and *CGB3–9* gene promoter regions. The differences were statistically significant (*p* < 0.01 and *p* < 0.001, respectively) (Fig. 2).

Statistical analysis of associations between methylation status of the promoters and expression of *CGB* genes did not show any significant correlations. Furthermore, methylation status of promoters for the analyzed genes did not correlate with stage or grade of the tumours.

### Expression of SP3 and TFAP2A transcriptional factors differs in control and cancerous ovaries

**Fig. 3:**
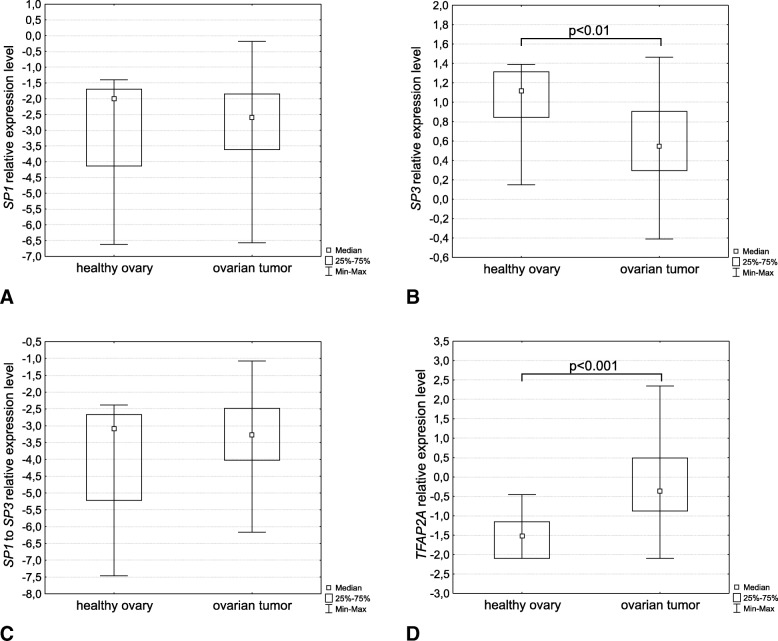
Relative expression level of (**a**) *SP1*, (**b**) *SP3*, (**c**) *SP1/SP3*, and (**d**) *TFAP2A* transcripts in healthy ovary and ovarian tumor tissue measured using RT-qPCR

The availability of transcription factors regulating *CGB* genes expression was assessed on RNA level with RT-qPCR. The assay, designed to quantify relative level of *SP1* transcripts, showed that the analyzed groups of samples did not differ in terms of expression of this transcription factor (Fig. 3a). However, ovarian tumours were characterized by significantly lower SP3 expression than control ovarian tissue (*p* < 0.01) (Fig. 3b). Nevertheless, *SP1* and *SP3* expression level ratio did not show significant differences between the two groups (Fig. 3c).

Significant differences between control and ovarian cancer tissues were seen for the expression of the *TFAP2A* gene, and was higher in case of ovarian cancer tissue (*p* < 0.001) (Fig. 3d).

Association analysis within the group of ovarian tumours showed that the level of* TFAP2A* transcripts correlated positively with *CGB3–9* gene expression (R = 0.67).

The expression level of these transcription factors did not correlate with the stage and grade of the tumours.

## Discussion

It has been known for some time that, not only gynecological cancers but also other tumours of many different origin are capable of secreting hCG and especially its free beta subunit. However, the regulation of *CGB* genes expression as well as the role of hCGβ protein in tumorigenesis have not been satisfactorily established.

It has been shown that both epigenetic and transcription factors might regulate the genes encoding human chorionic gonadotropin beta subunit. However, most of the studies regarding *CGB* expression have been conducted on trophoblasts cells or choriocarcinoma cell lines in vitro and not epithelial carcinomas.

In this study we have investigated the mechanisms regulating the expression of human chorionic gonadotropin beta subunit in non-trophoblastic cancer, specifically epithelial ovarian cancer tissues. For this purpose two complementary assays were adopted. First we analyzed epigenetic changes of *CGB* promoter fragments and in the next step we determined the expression levels of key transcription factors involved in the regulation of *CGB* expression. The data obtained were correlated with *CGB* mRNA levels. *CGB* genes expression on RNA level was analyzed in two groups. The first included *CGB1* and *CGB2* genes, the second consisted of the more transcriptionally active genes reported in placental studies: *CGB3/CGB9, CGB5, CGB6/CGB7* and *CGB8* [19, 27].

The results of our experiments demonstrated that both control and malignant ovarian tissues produce chorionic gonadotropin beta subunit transcripts. The expression of *CGB3–9* genes was not only confirmed for all studied samples, but was also shown to be significantly higher in cancer than control ovarian tissue. This is consistent with previous reports on the prevalence of the transcriptional activity of *CGB3–9* genes in malignant tissue when compared to benign changes or healthy tissues [19, 27]. *CGB1* and *CGB2* genes were detected to be transcriptionally active only in 20% of tested ovarian cancer tissues and at the lowest level across all tested *CGB* genes of the cluster. It is only recently, due to novel sensitive molecular techniques, that it has become possible to establish that these are active genes and not, as previously considered, pseudogenes [27–29]. In fact we previously demonstrated the presence of *CGB1* and *CGB2* transcripts in ovarian carcinomas [27]. Still, little is known about regulation of these genes expression and transcription factors assumed to regulate *CGB1* and *CGB2* expression were predicted using in silico analysis tools [30].

Associations between the expression of *CGB* genes and epigenetics have hitherto been studied only in normal placental tissue and it has been known for some time that the genome of placental tissue is characterized by a higher degree of hypomethylation compared to other healthy tissues; the *CGB* gene coding region is no exception to this [11–13, 31, 32]. Our previous study showed that *CGB3–9* are the most transcriptionally active *CGB* genes in placenta and are hypomethylated. However, so are *CGB1* and *CGB2* promoter regions. Here we have shown a similar pattern of hypomethylation and increased transcription of *CGB* genes in ovarian cancer tissue. Notably transcription of *CGB1* and *CGB2* was detected in 20% of ovarian cancer samples. Additionally, our experiments revealed that the degree of methylation of *CGB* promoter regions correlates with the level of *CGB* transcripts in chorionic tissue and placenta. Thus, epigenetic changes may be considered a factor contributing to pregnancy failure [13]. By implication it may be speculated that the induction of *CGB* gene cluster methylation could contribute to the decrease in hCGβ expression by tumours, which may be advantageous to cancer patients.

The manner in which epigenetics influences carcinogenesis varies among different carcinomas. However, DNA hypomethylation or demethylation was the initial epigenetic aberration recognized in human tumours [33] and is still extensively studied as a phenomenon associated with the development and survival of cancer cells. In this context our results show that ovarian cancers are characterized by demethylation of the *CGB* genes compared to control ovary tissues.

There were no significant correlations between methylation status and expression level of particular *CGB* genes. This fact might be the consequence of the adopted methods. Even though RT-qPCR is a highly sensitive and accurate method, methylation specific PCR (MSP) is a semi-quantitative technique. Thus, combining data obtained using these two methods in the statistical analysis might not have yielded significant differences, although differences between healthy ovary and cancerous tissue were evident in both assays.

It has been shown, that for *CGB* genes transcription in placenta, the most important seems to be 360 nucleotides long 5’UTR region with binding sites of transcription factors such as: SP1, SP3 and AP2, which regulate basal *CGB* expression [13, 22, 26, 34, 35]. Our previous results revealed strong correlations between *TFAP2A* transcripts and *CGB1–9* as well as *CGB3–9* mRNA levels in term placenta [13]. In the present study conducted on cancer tissue, we confirm the significance of *TFAP2A,* as its higher expression in cancer tissues in contrast to normal ovary was noted. What is more strong positive correlation between the amount of the factor’s mRNA and that of *CGB3–9* in this group has been shown.

The influence of *SP1* and *SP3* expression on *CGB* expression in cancer is very difficult to assess. The results showed that healthy ovaries and ovarian cancers did not differ in terms of *SP1* transcription factor expression. On the other hand, ovarian tumours were characterized by significantly lower level of *SP3* transcripts. The expression of these transcription factors did not correlate with *CGB* transcriptional activity. Still, it is known that SP1 and SP3 are factors which can extend opposite effects on *CGB* genes expression in trophoblast. Therefore, interpretation of the results obtained for cancers may not be so straightforward [22, 26]. Nevertheless, it cannot be excluded that similar mechanisms may regulate *CGB* gene expression during both pregnancy and in cancer.

Parallels between pregnancy development and cancer have been shown before. Malignant and trophoblastic cells share the ability to escape apoptosis, migrate and invade surrounding tissues while evading a host immune response [36]. Because of a common mechanisms influencing *CGB* genes expression in pregnancy and cancer shown in our studies, it can be expected that regulation of these processes might share some common features. Indeed, the results of the present study showed that, similar to the placenta, the expression of *CGB* genes in ovarian cancer strongly correlates with *TFAP2A* expression*.* Additionally both tissues are characterised by the demethylation of *CGB* promotors.

Further studies on the common pathways of *CGB* genes transcription regulation in trophoblast and malignant cells can lead to the development of novel diagnostic approaches for the early identification of both pathologies in pregnancy and cancer.

## Conclusions

In ovarian cancers increased expression of human chorionic gonadotropin beta subunit is associated with demethylation of *CGB* promoter regions and *CGB3–9* expression level strongly correlates with expression of *TFAP2A* transcription factor. This seems to point to a common mechanism of *CGB* expression regulation in placenta and ovarian cancer. Furthermore presence of mRNA arising from *CGB1* and *CGB2* genes appears to be a unique feature of a subset of ovarian cancers.

## Data Availability

All data generated or analyzed during this study are included in this published article and on the website: http://www.katbiolkom.ump.edu.pl/wp-content/uploads/repozytorium/RAW-DATA_J-Ovarian-Research_25072018.xlsx

## Abbreviations

5’UTR: 5′ untranslated region
AP2: Activating enhancer binding protein 2 alpha
BBQ: BlackBerry Quencher
*CGB*: human chorionic gonadotropin beta subunit gene
CGB1–2_ M: primers complementary to the methylated variant of *CGB1–2* genes
CGB1–2_ NM: primers complementary to the non-methylated variant of *CGB1–2* genes
CGB3–9_ M: primers complementary to the methylated variant of *CGB3–9* genes
CGB3–9_NM: primers complementary to the non-methylated variant of *CGB3–9* genes
*DAPK1*: Death associated kinase 1 gene
ETS-2: protein C-ets-2
hCG: human chorionic gonadotropin
hCGβ: human chorionic gonadotropin free beta subunit
*HPRT*: hypoxanthine-guanine phosphoribosyltransferase 1
MSP: methylation specific polymerase chain reaction
MTA3: metastasis-associated protein
OCT3/4: octamer-binding transcription factor 3/4
P53: tumor suppressor p53
PPARγ: peroxisome proliferator-activated receptor gamma
RT-qPCR: Reverse transcriptase quantitative polymerase chain reaction
SP1: Specificity protein 1
SP3: Specificity protein 3
*TFAP2A*: transcription factor AP2 gene
UICC: The Union for International Cancer Control
